# The psychometric properties of the Italian adaptation of the Health Orientation Scale (HOS)

**DOI:** 10.1186/s12955-020-01298-z

**Published:** 2020-03-13

**Authors:** M. Masiero, S. Oliveri, I. Cutica, D. Monzani, F. Faccio, K. Mazzocco, G. Pravettoni

**Affiliations:** 1 Department of Biomedical and Clinical Sciences, University of Milan, Via Festa del Perdono 7, Milan, Italy; 2 Applied Research Division for Cognitive and Psychological Science, European Institute of Oncology, IRCSS, Milan, Italy; 3 Department of Oncology and Hemato-Oncology, University of Milan, Milan, Italy

## Abstract

**Background:**

A novel approach suggested that cognitive and dispositional features may explain in depth the health behaviors adoption and the adherence to prevention programs. The Health Orientation Scale (HOS) has been extensively used to map the adoption of health and unhealthy behaviors according to cognitive and dispositional features. Coherently, the main aim of the current research was to assess the factor structure of the Italian version of the HOS using exploratory and confirmatory factor analysis and testing the construct validity of the scale by assessing differences in health orientations between tobacco cigarette smokers and nonsmokers.

**Method:**

The research protocol was organized in two studies. *Study 1* evaluated the dimensionality of the HOS in a sample of Northern Italian healthy people. Three hundred and twenty-one participants were enrolled; they were 229 women (71.3%) and 92 men (28.7%). In *Study 2*, the factor structure and construct validity of the HOS Italian version was assessed trough confirmatory factor analysis using a tobacco cigarette smokers and nonsmokers population. Two hundred and nineteen participants were enrolled; they were 164 women (75.2%) and 55 men (24.8%).

**Results:**

In *Study 1*, a seven factors solution was obtained explaining 60% of cumulative variance instead of 10 factors solution of the original version of the HOS. In *Study 2,* the factor structure of the Italian version of the HOS was confirmed and applied to the smokers and nonsmokers; nonsmokers reported higher values than smokers in Factor 1 (MHPP) [t (208) = − 2.739 *p* < .007] (CI 95–4.96% to −.809), Factor 2 (HES) [t (209) = − 3.387 *p* < .001] (CI 95–3.93% to -. 1.03), Factor 3 (HIC) [t(213) = − 2.468 *p* < .014] (CI 95–2.56% to −.28) and Factor 7 (HEX) [t(217) = − 3.451 *p* < .001] (CI 95%- 1.45 to .39).

**Conclusions:**

Results of the Italian adaptation of HOS lead to a partial redistribution of items and confirmed 7 subscales to distinguish psycho-cognitive dispositional dimensions involved in health orientation styles.

## Background

The Health-Related Quality of Life Program developed by the Center for Disease Control and Prevention (CDC) focuses on how well-being can be integrated into health promotion and measured in public health surveillance (https://www.cdc.gov/hrqol/wellbeing.htm). According to recent evidence, well-being and health status largely depend on our daily behaviors such as physical exercise, healthy eating, and avoiding risk behaviors (e.g., smoking or alcohol consumption and so on) [1–4]. Moreover, the integration of regular screening for early detection of diseases along with the implementation of guidelines directed at the avoidance of risk factors, might limit the effects of the major causes of morbidity and mortality [5]. Nevertheless, most people find it difficult to adopt healthy habits or to decide to follow specific preventive or prophylactic programs.

A novel approach suggested that cognitive and dispositional features may explain in depth the health behaviors adoption and the adherence to prevention programs [6, 7]. For example, the five-factor model that described the personality according to five dimensions (*openness*, *conscientiousness*, *extraversion*, *agreeableness*, and *neuroticism*) conveyed that highly conscientious individuals (people characterized by the attitude to be organized, reliable and deliberative, and/or to have a high sense of competence, duty and need for achievement) are more likely to wear seat belts, to do physical exercise regularly, to get enough sleep, and to consume more fruits and vegetables [8–10]. They are also less likely to smoke cigarettes, to consume alcohol and binge drink. In particular, the role of psycho-cognitive and dispositional aspects in the modulation of health behaviors was investigated in depth for tobacco cigarette smoking [11–15]. Accruing evidence on psycho-cognitive mechanisms reported that smokers tend to underestimate the risk to incur in smoking related disease (for example, lung cancer and emphysema [16, 17], when they compare themselves to other smokers with the same characteristics (age, number of cigarette per day, years of smoking and so on). Smokers usually judge their health status better than nonsmokers [18], showing an alteration in risk perception, and they use cigarettes as emotional self-regulation strategy [19]. Furthermore, smokers may use the cigarettes to increase their attentional level and/or to improve their performance before of an examination, or, to reduce and modulate anxiety, worry, and depression (for example, the impulsivity and anhedonia are positively associated with smoking [20]. Otherwise, studies on personality observed that dispositional factors seem to affect the decision to start, to maintain and to quit [21, 22]. For instance, Hakulinen and colleagues (2015) reported that personality traits as “higher extraversion” (refers to people characterized by the proneness to emotional instability, anxiety and depression) and “lower conscientiousness” were positive related to the smoking initiation [23]. In similar way, the conscientiousness seemed to safeguard besides to the smoking persistence [24]. Even the construct of the control has a pivotal role in the modulation of smoking behaviours. Smokers tend to believe to have a good control of their number of daily cigarettes or to quit easily, and this is an important roadblock for smoking cessation [16, 25]. After several attempts to give up, they recognize the difficulty to cope with physical and psychological dependence, and they tend to relapse. Often, smokers tend to attribute to fate the occurrence of negative events and diseases (*external health control*), while nonsmokers are more prone to recognize the role of their behaviours in their health status (*internal locus of control*) [26].

Many decades ago, Snell and colleagues (1991) [27] addressed the issue of assessing personality traits which can influence health behaviors and promote well-being developing a tool named the Health Orientation Scale (HOS). HOS has been extensively used to map the adoption of health and unhealthy behaviors [28–32]. It is composed of 50 items evaluated on a five points Likert scale (from “*Not all characteristic of me*” to “*Very characteristic of me*”) concerning personality tendencies that can be associated with health behaviors, contribute to improve well-being and influence decision-making processes about the health. Items are divided into 10 different subscales with five items for each scale. The authors do not assume an explicit theoretical model for building this scale, but they aimed at unifying in a single instrument a few meaningful constructs from different theoretical areas: self-efficacy, cognitive-emotional factors, motivation toward health, perceived control.

Self-efficacy refers broadly to the theories of Albert Bandura [33, 34]. Two HOS scales belong to this construct: *Personal Health Consciousness* (PHC) and *Health-Esteem and Confidence* (HEC). PHC is defined as the dispositional tendency to spend time thinking about one’s physical health and fitness [27]. Gould (1988) considered health consciousness as a psychological or inner status, including health alertness, health self-consciousness, health involvement, and health self-monitoring [35].

According to Gould [35, 36], health consciousness is a psychographic variable that is not integrated with visible behaviors. Therefore, measures of attitude and behavior regarding health care and prevention as dependent variables are predicted by health consciousness as an independent variable. According to Iversen and Kraft [37], health consciousness is also defined as “the tendency to focus attention on one’s health” (p. 603) and differs from health anxiety or fear of being sick. Iversen and Kraft found a positive correlation between health consciousness and preventive health behavior (e.g., fruit and vegetable consumption and exercise).

Self-esteem is strongly linked to health-related behaviors. For example, people with higher levels of self-esteem are more likely to engage in behaviors that protect and maximize health [38–40]. The HEC subscale concerns the tendency to feel sure, positive and confident about the physical status and psychophysical well-being in general. People who endorse these items are confident that their health is robust and durable. They are oriented to keep in control of their own health, wellness and get advantage of healthcare progresses [27]. These dimensions should not be confused with positive affects (e.g. optimism) towards ones’ own health, which depend largely on cognitive factors such as social comparison processes [41].

The HOS even includes one measure of social comparison: the *Health Image Concern* (HIC). This is the chronic tendency to be aware of the external, observable impression that one’s physical health makes on others [27]. People who have high scores in health image concern are strongly worried about the public impression created by their fitness. The HIC creates dissatisfaction or persistent and chronic distress, which can affect self-worth and involve several areas of concern [42–44].

Also cognitive constructs and emotional aspects were taken into account by the authors developing the tool: *Health Anxiety* (HA), *Health Expectations* (HE) and *Health Status* (HS). HA addresses worries about one’s physical health [45]. According to the cognitive-behavioral theories it could be related to an enduring tendency to negatively misinterpret bodily variations and other ambiguous health-related information, including the results of medical consultation [46, 47]. Watson and Pennebaker [48] found a strong correlation between negative emotions and evaluation of your own health status, and between negative emotions and health expectations. Both these dimensions are assessed by the HOS scale. HE refers to the tendency to feel positive (or negative) about your own future physical health. It measures people’s belief that their well-being and their future physical health will continue to be good (or bad). HS refers to a current evaluation of your own physical and psychological status: people who endorse these items believe that they are in excellent psychophysical health.

Although there are several theories on health motivation [49] the nature of this factor and how it can affect health behaviors have been poorly studied in literature, giving almost for granted that people by nature are all equally motivated to preserve their health. Nevertheless, it is clear, that a large number of individuals have serious difficulties engaging in health-promoting behaviors despite their high motivation. For example, about 68% of smokers in 2015 reported the intention to quit completely [50], but the average number of quit attempts taken before quitting successfully is usually very high [51], while the rate of success is still poor [52]. In addition, people who are highly motivated to know their genetic make-up do not translate risk information into life style changes [53, 54]. Other people are simply unmotivated to engage in health-related behaviors, for example, up to 30% of individuals express no intention to exercise [55]. Snell and colleagues [27] measured motivation toward health through two scales: *Motivation to Avoid Unhealthiness* (MAU) and *Motivation for Healthiness* (MFH). MAU refers to the tendency to avoid being or becoming unhealthy, to avoid poor physical health, to avoid behaviors and activities, which undermine physical health. MFH refers to the motivation to pursue positive physical health and to maintain excellent physical health. Individuals who endorse these items are motivated to engage in activities that promote their physical health and to strive to preserve their well-being and integrity of their physical health.

Finally, Snell and colleagues also included a measure of the beliefs regarding the perception of control over your own health: the *Health Internal Control* (IHC) and the *Health External Control* (EHC). Health locus of control is the extent to which individuals attribute their health status to their own actions or to environmental circumstances and powerful external agents [56]. People who show an IHC tend to believe that their health status is determined by their own personal control, thus they can exert an influence on their health based on their decisions and actions. On the contrary, people who show an EHC are marked by their belief of the influence of fate, powerful others, or supernatural occurrences upon one’s health, which they themselves can neither anticipate nor influence. Moreover, they believe that being in good or bad health depends on the behavior of health specialists (doctors, nurses, therapists) or significant others (family, relatives, friends). These individuals tend to perceive that their health is outside of their personal control. Studies based on larger samples found expected associations between locus of control and healthy behaviors [57–59] or risk behaviors. For instance external locus of control was associated with smoking relapse, or with less physical activity and less attention to healthy nutrition [60–62].

Considering this theoretical background and HOS utility in measuring psycho-cognitive and dispositional tendencies toward health, the main aims of the current study were two. Firstly, to assess the HOS factorial structure using an EFA methods and a parallel analysis in order to identify the number of factors that might be extracted and retained in Italian adaptation. This is first study on Italian speaking population. Although a long time has passed since its publication, to date no study performed a factorial analysis and accurately validated this tool. Originally, in the protocol developed by Snell and colleagues, 10 dispositional factors were identified. The analyses were conducted checking the internal consistency of each subscales using Cronbach alpha coefficient and correlational analysis. Notwithstanding, Snell and colleagues limited their analysis without checking for a possible reduction of the factors that could be more explanatory or predictive of behavioral changes.

Secondly, to replicate the factor structure through Confirmatory Factor Analysis (CFA) and investigate the construct’s validity in a sample of Italian tobacco cigarette smokers and nonsmokers. The analysis on smokers and nonsmokers allowed assessing the capacity of the HOS Italian adaptation to distinguish dispositional and cognitive dimensions involved in health orientation styles. Coherently to the accruing evidence reported, we hypothesized that smokers and nonsmokers have different psycho-cognitive traits that may affect adoption and maintenance of unhealthy behaviours. This second study is particularly innovative, since tried to integrate all the evidence collected on smoking behaviours [11–13, 20, 24], and personality traits using a standardized tool like the Italian version of HOS.

## Study 1

The aim of the first study was to evaluate the dimensionality of the Italian version of the HOS by performing an exploratory factor analysis (EFA).

### Material and method

#### Participants

The Italian adaptation of the HOS was tested in Northern Italy using a non-clinical population. Data were collected using a convenience sample recruited through social networks, University of Milan mailing lists and authors’ acquaintances. A sample of 321 participants (229 women, 71.3%, and 92 men, 28.7%), was enrolled. They had a mean age of 31.36 (SD = 7.96; min = 16; max = 62). The educational level of the sample enrolled was distributed as follows: doctoral degree (7.8%), specialization (15.8%), master degree (34.9%), bachelor (29%), high school (11.8%), and elementary school (0.3%). We have not information about educational level for 0.3% of participants.

#### Recruitment

Data collection procedure was conducted from January 2016 to June 2016. The questionnaire was administered by means of Lime Survey, an on-line platform (https://www.limesurvey.org/), which is able to register the computer ID number of participants ensuring they filled in the questionnaire once. Participation to the study was voluntary, and in each moment, participants could decide to withdraw. Invitation to fill in the questionnaire was associated with a brief letter of presentation about the validation study. Each participant was provided with an informed consent that had to be signed. The study was in accordance with the principles stated in the Declaration of Helsinki (59th WMA General Assembly, Seoul, 2008).

#### Measures

Health Orientations: The back translation method was used to create the Italian version of the HOS. Consequently, the questionnaire was translated from English to Italian by an Italian mother tongue (Version 1), and then translated back from Italian to English (Version 2) by another independent English mother tongue. Finally, a third translator compared and encompassed Version 1 and Version 2. This action allowed to overcome disagreement and to obtain a final version of the questionnaire.

Demographic characteristics: a set of items assessed participants’ socio-demographic variables (gender, age, educational level) and physical variables (height and weight).

#### Data analysis

EFA was conducted to determine the factorial structure of the Italian adaptation of HOS. The extraction method applied was the principal-axis factor extraction followed by a promax (oblique) rotation. The results of Kaiser-Meyer-Olkin (KMO) values and Bartlett’s Test of Sphericity were examined to test the eligibility of the HOS in factor extraction. Value of KMO index of 0.873 (meritorious; [63]) and significance of the Bartlett’s Test of Sphericity [*Χ*^2^(1225) = 6876.39, *p* < .001] indicated that the data was factorable and that EFA could be performed on responses of the Health Orientation Scale.

Parallel analysis (PA) were implemented prior to perform the EFA in order to determine the exact number of factors to retain [64]. PA is the most recommended procedure to select the optimal number of factors in the data [65–67]. Specifically, this statistical technique allows to reduce over identification of factors due to sampling error [68]. Factors with eigenvalues above of 95th percentile of the eigenvalues of the parallel factor were retained. Subsequently, item retention was based on two main criteria: primary factor loading and secondary factor loading. More in details, we retained items that reported a primary saturation > ǀ3.5ǀ and a ratio between primary and secondary saturation > ǀ1.5ǀ. Cronbach alpha coefficient was used to assess the internal consistency of each factor. Correlational analysis was performed to assess association between the identified factors. All the analyses were performed with the SPSS package (version 20.0, IBM).

#### Ethics approval

Ethics approval was not request by the Institution where the project was conducted due to the study method (on-line survey). Also, the participation was volunteer and anonym, in each moment, they might withdraw their initial consent. Each participant received a details presentation of the research before to collect the data. Finally, the study had not the risk to develop adversely affect the physical and mental of the subjects consisting in a set of items that investigated attitude to health behaviors.

### Results

Kurtosis and skewness were checked for all variables. They were all below 2 in absolute value and thus denoting a normal distribution.

The PA performed on all 50 items of the HOS retained nine factors. The first factor accounted for 23.5% of the variance (eigenvalue = 11.77), while the last factor explained 2.3% of the variance (eigenvalue = 1.13). All nine factors accounted 61% of the cumulative variance.

According to criteria reported above, items 27, 40 and 8, 28 were removed because they did not respect primary factor loading > ǀ3.5ǀ, while items 24, 1, 50 and 29 were removed because they did not respect ratio between primary and secondary factor loading >ǀ1.5ǀ.

After the elimination of the item 29, the ninth factor was not loaded by any item. For this reason, a new PA was implemented in order to check the exact number of factors to be extracted in subsequent analysis. This PA indicated to retain 8 instead of 9 factors of the initial solution. The first factor accounted for 24% of the variance (eigenvalue =10.29), while the last factor explained 2.5% of the variance (eigenvalue = 1.10). All eight factors accounted for 61% of the cumulative variance.

Then, we proceeded to check primary and secondary factor loading. Items 18, 7, 46 and 30 were removed because they did not respect the ratio between primary and secondary saturation > ǀ1.5ǀ. After the deletion of item 30, the factor 8 was not loaded by any item. We then decided to perform a new PA that confirmed to retain a solution with 7 factors instead of a solution with 8 factors. The first factor accounted for 23.1% of the variance (eigenvalue =9.015), while the last factor explained 3.3% of the variance (eigenvalue = 1.29). All seven factors accounted for 60% of the cumulative variance. We then removed items 34 and 6 because they did not respect ration between primary and secondary factor loading. The final factor solution is displayed in Table 1. A total of 36 items composed the final version of the questionnaire.

- **Factor 1**: was composed by items 5, 15, 25, 35, 45, 16, 26, 36 and 33. The Cronbach alpha coefficient was 0.882 (good). Based on the meaning of these items, the first factor was labelled *Motivation for health promotion and prevention (MHPP).*
- **Factor 2:** Health esteem (HES).
  It was composed by items: 4, 14, 44, 9, 19, 10 and 20. In this factor were embedded items from Health esteem and confidence, Health expectation and Health status subscales The Cronbach alpha coefficient was 0.838 (good).
- **Factor 3**: Health Image Concern (HIC).
  It was composed by items: 2, 12, 22, 32 and 42. This factor reproduced the original subscale named the Health Image Concern. The Cronbach alpha coefficient was 0.832 (good).
- **Factor 4**: Personal health consciousness (PHC).
  It was composed by items: 11, 21, 31 and 41. This factor substantially reproduced the original subscale named Personal Health Consciousness. The Cronbach alpha coefficient was 0.822 (good).
- **Factor 5:** Health locus of control (HLC).
  It was composed by items: 17, 37, 47, 38 and 48. In this factor were embedded items from the Health Internal Control and the Health External Control subscales. The Cronbach alpha coefficient was 0.77 (acceptable).
- **Factor 6:** Health anxiety (HA).
  It was composed by items: 3, 13, 23 and 43. This factor reproduced the original subscale named Health Anxiety without item 33. The Cronbach alpha coefficient was 0.797 (acceptable).
- **Factor 7:** Health Expectations (HEX).
  It was composed by items 39 and 49. This factor reproduced the original subscale named Health expectation, without items 9, 19 and 29. The Cronbach alpha coefficient was 0.716 (acceptable).
  Table 2 reports Pearson correlation values among the seven identified factors. As shown, these correlations ranged from .15 to .51 (see Table 2).

**Table 1:** Motivation for health prevention and promotion (MHPP); Health esteem (HES); Health image concern (HIC); Personal health consciousness (PHC); Health Locus of control (HLC); Health anxiety (HA); Health expectation (HEX)

Item HOS	MHPP *Factor 1*	HES *Factor 2*	HIC *Factor 3*	PHC *Factor 4*	HLC *Factor 5*	HA *Factor 6*	HEX *Factor 7*
**5. EN**: *I do things that keep me from becoming physically unhealthy.***IT**: *“Faccio cose che mi preservano dalle malattie fisiche”.*	**0.47**	0.16	-0.05	0.13	0.01	0.04	-0.05
**15.***I am motivated to keep myself from becoming physically unhealthy.***IT**: *Sono molto motivato ad evitare di ammalarmi fisicamente.*	**0.56**	0.10	-0.09	0.09	0.06	0.18	0.06
**25. EN**: *I try to avoid engaging in behaviors that undermine my physical health.***IT***: Cerco di evitare di assumere comportamenti che possano compromettere la mia salute fisica.*	**0.63**	0.06	-0.20	0.02	-0.02	0.09	-0.03
**35. EN**: *I really want to prevent myself from getting out of shape.***IT**: *Desidero molto evitare di essere fuori forma.*	**0.78**	-0.11	0.13	-0.12	0.06	-0.12	-0.02
**45***.***EN**: *I am motivated to avoid being in terrible physical shape.***IT**: *Sono molto motivato ad evitare di essere in pessima forma fisica.*	**0.52**	0.13	-0.01	0.01	0.11	0.10	-0.10
**16***.***EN***:I am strongly motivated to devote time and effort to my physical health.***IT**: *Sono fortemente motivato a dedicare tempo e sforzi alla mia salute fisica.*	**0.74**	-0.06	-0.05	0.01	0.07	0.06	-0.15
**26***.***EN**: *I have a strong desire to keep myself physically healthy.***IT***: Ho un forte desiderio di mantenere la mia salute fisica.*	**0.82**	0.05	-0.12	-0.04	-0.14	0.09	-0.00
**36***.***EN**: *It's really important to me that I keep myself in proper physical health.***IT**: *E’ molto importante per me mantenermi in buona salute fisica.*	**0.81**	-0.02	0.12	-0.03	-0.05	-0.11	-0.06
**33. EN**: *I usually worry about whether I am in good health.***IT**: *Di solito mi preoccupo di essere o meno in buona salute.*	**0.44**	-0.10	0.09	0.16	0.06	0.15	0.04
**4. EN**: *I feel confident about the status of my health.***IT**: *Mi sento sicuro di essere in salute.*	0.02	**0.55**	0.06	-0.02	0.00	-0.26	0.07
**14. EN**: *I rarely become discouraged about my health.***IT**: *Difficilmente mi scoraggio pensando alla mia salute.*	0.12	**0.46**	-0.05	0.13	0.09	-0.17	0.15
**44. EN**: *I feel that I have handled my health very well.***IT**: S*ento di aver preservato molto bene la mia salute.*	0.15	**0.60**	-0.02	0.02	-0.06	0.03	0.00
**9. EN**: *I expect that my health will be excellent in the future.***IT**: *Mi aspetto che la mia salute sarà eccellente nel futuro.*	-0.08	**0.71**	0.05	0.01	0.07	0.17	-0.19
**19. EN**: *I believe that the future status of my physical health will be positive.***IT**: *Credo che la mia saluta fisica sarà buona in futuro.*	-0.15	**0.75**	0.02	0.04	0.14	0.05	-0.12
**10***.***EN**: *I am in good physical health.***IT**: *Sono in buona salute fisica.*	0.15	**0.72**	0.03	-0.11	-0.08	-0.15	0.15
**20***.***EN**: *My body is in good physical shape.***IT**: *Il mio corpo è in* *buona forma fisica.*	0.29	**0.60**	0.03	0.00	-0.15	-0.123	0.07
**2. EN**: *I sometimes wonder what others think of my physical health.***IT**: *Qualche volta mi chiedo cosa gli altri pensino della mia salute fisica.*	-0.04	-0.02	**0.64**	0.08	0.04	0.08	-0.07
**12. EN**: *I’m very concerned with how others evaluate my physical health.***IT**: *Sono molto preoccupato di come gli altri giudicano il mio stato di salute fisica.*	0.02	-0.04	**0.85**	0.10	-0.09	0.03	-0.09
**22. EN**: *I'm very aware of what others think of my physical health.***IT**: *Sono molto consapevole di cosa gli altri pensino del mio stato di salute fisica.*	0.01	0.02	**0.49**	0.14	0.05	0.04	-0.14
**32. EN**: *I'm concerned about how my physical health appears to others.***IT**: *Sono preoccupato di come la mia salute fisica appare agli altri.*	0.01	0.02	**0.84**	-0.09	-0.05	0.05	-0.01
**42. EN**: *I'm concerned about what other people think of my physical health.***IT**: *Sono molto preoccupato di cosa le altre persone pensano della mia salute fisica.*	-0.13	0.13	**0.73**	-0.05	-0.06	0.14	0.06
**11. EN**: *I notice immediately when my body doesn't feel healthy.***IT**: *Mi accorgo subito quando il mio corpo non si sente in buona salute.*	-0.05	0.06	0.04	**0.80**	0.03	-0.10	-0.03
**21. EN**: *I'm sensitive to internal bodily cues about my health.***IT**: *Sono sensibile ai segnali interni del mio corpo come indici di salute.*	0.21	0.02	-0.04	**0.57**	-0.04	0.02	0.14
**31*.*EN**: *I know immediately when I'm not feeling in great health.***IT**: *Mi accorgo immediatamente quando non sono in buona salute.*	0.05	0.00	0.01	**0.74**	-0.03	0.03	-0.05
**41. EN**: *I'm very aware of changes in my physical health.***IT**: *Sono molto consapevole dei cambiamenti nella mia salute fisica.*	-0.06	-0.03	0.10	**0.80**	-0.00	-0.07	0.10
**17**. **EN**: *My health is something that I alone am responsible for.***IT**: *La mia salute è qualcosa di cui solo io sono responsabile.*	-0.02	0.16	-0.01	-0.06	**0.56**	0.14	-0.10
**37.***.***EN**: *What happens to my physical health is my own doing.***IT**: *Ciò che capita alla mia salute fisica è la conseguenza del mio agire.*	0.09	-0.01	0.18	0.06	**0.59**	-0.13	0.14
**47. EN**: *Being in good physical health is a matter of my own ability and effort.***IT**: *Il mio essere in buona salute fisica dipende dalla mia abilità e dal mio sforzo.*	0.22	-0.01	0.07	-0.10	**0.72**	-0.15	0.13
**38. EN**: *Being in excellent physical shape has little or nothing to do with luck***IT**: *Godere di un’eccellente forma fisica ha poco o per nulla a che fare con la fortuna.*	-0.03	-0.02	-0.08	0.01	**0.58**	-0.03	0.01
**48. EN**: *I don't believe that chance or luck play any role in the status of my physical health.***IT**: *Non credo che il caso o la fortuna* *influenzino il mio stato di salute fisica.*	-0.10	-0.04	-0.17	0.03	**0.67**	**0.12**	-0.10
**3. EN**: *I feel anxious when I think about my health.***IT**: *Mi sento ansioso quando penso alla mia salute.*	0.12	-0.11	-0.00	0.06	-0.05	**0.64**	0.11
**13. EN**: *I'm worried about how healthy my body is.***IT***: Sono preoccupato del mio stato di salute fisica.*	0.15	-0.15	0.17	0.08	0.04	**0.44**	0.06
**23. EN**: *Thinking about my health leaves me with an uneasy feeling.***IT**: *Pensare alla mia salute mi lascia con una sensazione di disagio.*	0.06	-0.06	0.11	-0.11	0.000	**0.59**	0.22
**43***.***EN**: *I feel nervous when I think about the status of my physical health.***IT**: *Mi sento nervoso quando penso al mio stato di salute fisica.*	-0.04	0.06	0.18	-0.14	0.041	**0.72**	0.10
**39. EN**: *I will probably experience a number of health problems in the future.***IT**: *Probabilmente avrò alcuni problemi di salute in futuro.*	-0.13	0.08	-0.09	0.02	-0.00	0.28	**0.65**
**49. EN**: *I anticipate that my physical health will deteriorate in the future.***IT**: *Prevedo che la mia salute fisica peggiorerà in futuro.*	-0.11	-0.01	-0.11	0.08	0.013	0.16	**0.67**

*p < 0.05, **p < 0.01, ***p < .001

**Table 2:** In the table, all correlations between factors of the HOS are reported

Italian adaptation HOS	MHPP	HES	HIC	PHC	HLC	HA	HEX
MHPP	.						
HES	.509**	.					
HIC	.006	−.020	.				
PHC	.506**	.328**	−.014	.			
HLC	.307**	.251**	.178**	.148*	.		
HA	.090	−.257**	.465**	.103	.826	.	
HEX	.069	.271**	−.110	−.162**	−.015	−.379**	.

*p < 0.05, **p < 0.01, ***p < .001

Motivation for health promotion and prevention (MHPP); Health esteem (HES); Health image concern (HIC); Personal health consciousness (PHC); Health locus of control (HLC); Health anxiety (HA); Health expectation (HEX)

Results showed that some subscales strongly correlated with each other, such as MHPP positively correlated with HES, PHC and HLC. Snell and colleagues reported similar results about subscales correlations (Snell et al., 1991). Interestingly Health Anxiety (HA) positively correlated with the Health Image Concern (HIC) and negatively correlated with the Health Expectation (HEX) or with Health Esteem and confidence (HEC), showing that higher anxiety toward the health corresponds to higher worry about the public impression of physical status, scarce self-esteem, but reduces the expectation to experience health problems in the future. Nevertheless, some correlations among the Italian adaptation of HOS subscales were not significant instead, e.g. Motivation for health promotion and prevention (MHPP) with Health Image Concern (HIC), Health Anxiety (HA) and Health Expectation (HEX), suggesting that these subscales explain independent attitudes toward health.

## Study 2

The aim of this second study was to replicate the factor structure of the HOS by performing CFA and test its construct’s validity in a sample of tobacco cigarette smokers and nonsmokers. The analysis on smokers and nonsmokers allowed to investigate the capacity of the HOS Italian adaptation to distinguish dispositional and cognitive dimensions involved in health orientation styles.

### Material and method

#### Participants

Two hundred and nineteen participants (164 women, 75.2%, and 55 men, 24.8%) were enrolled. They had a mean age of 31.25 (SD = 8.67; min = 18; max = 62). The nonsmokers group involved 130 subjects (59.9%) who had never smoked during their lifetime, whereas smokers group involved 88 subjects (40.1%) who engaged in smoking behaviours in their lifetime. The mean age for the first cigarette was 16.77 (SD = 2.76), while the mean dependence level was 1.50 (SD = 2.064) assessed by the Fagerstrom Nicotine Dependence Test [69]. Educational level was distributed as follows: specialization (20.2%), master degree (34.9%), bachelor (37.2-%), high school (7.3%). We have not information about educational level for 0.4% of participants.

#### Recruitment

The HOS was administered to a convenience sample of Italian tobacco cigarette smokers and nonsmokers. Data collection was conducted from October 2016 to December 2016. Each participant received an e-mail containing three documents: a full explanation of the research protocol (goals and methodology), the informed consent and a link to fill in the HOS questionnaire. For this phase the same Limesurvey online platform used for HOS validation (https://www.limesurvey.org/) was used. Participation to the study was voluntary and in each moment participants could decide to withdraw. The study was in accordance with the principles stated in the Declaration of Helsinki (59th WMA General Assembly, Seoul, 2008).

#### Ethics approval

Ethics approval was not request by the Institution where the project was conducted due to the study method (on-line survey). Also, the participation was volunteer and anonym, in each moment, they might withdraw their initial consent. Each participant received a detailed presentation of the research before collecting the data. Finally, the study had not the risk to develop adversely affect the physical and mental of the subjects consisting in a set of items that investigated attitude to health behaviors.

#### Measures

*Demographic characteristics*: a set of items assessed participants’ socio-demographic variables (gender, age, educational level) and physical variables (height and weight).

*Health Orientations:* was assessed with the Italian version of the HOS.

*Smoking status definition*: a set of items was used to assess current participant’s smoking habits and past experience with smoking behaviours. In particular, the smoking status was assessed asking to each participant if he/she smoked in the last 30 day. The item was taken from the study conducted by Arnett (2000) and adapted by Masiero and colleagues [17, 70]. Each smoker completed a set of items on their smoking career, which included questions concerning smoking initiation, number of cigarettes per day, and number of years as smokers.

*Fagerstrom Test for Nicotine Dependence (FTND):* it is a 6-item self-administered questionnaire assessing nicotine dependence. The score range is from 0 to 10 points. It allows to split the nicotine dependence in four categories: low dependence (0–2); middle (3–4); strong (5–6); very strong (7–10) [71].

### Results

CFA with robust maximum likelihood (MLR) [72] was performed with Mplus 8.2 to evaluate the factor structure of the HOS identified in Study 1. Overall goodness-of-fit of the HOS factor structure model was evaluated assessing multiple indices of fit: the chi square test (χ^2^), the root mean square error of approximation (RMSEA), the comparative fit index (CFI), and the standardized root mean square residual (SRMR). The model fit was firstly evaluated using the χ^2^ statistic. However, because of its sensitivity to the sample size, other indices were also used [73]. Specifically, values above .90 for the CFI, a RMSEA below .06, and a SRMR below .08 indicate a good fitting model. Because the rate of missing responses at the HOS was negligible (i.e., 9.2% of participants did not answer to one item, .5% at two, 1.8% at three, and .5% at four items), missing responses at the HOS were handled using a robust full information maximum likelihood (FIML) estimation procedure. The factor structure of the HOS displayed a good fit to data [SB χ^2^ (573) = 930.108, *p* = .000; RMSEA = .053; CFI = .901; SRMR = .064]. As showed in Table 3, all standardized factor loadings were significant and greater than .531.

**Table 3:** Results of the CFA with standardized factor loadings (and standard errors) of the HOS

Item HOS	MHPP *Factor 1*	HES *Factor 2*	HIC *Factor 3*	PHC *Factor 4*	HLC *Factor 5*	HA *Factor 6*	HEX *Factor 7*
**5.***I do things that keep me from becoming physically unhealthy.*	.710 (.042)***						
**15.***I am motivated to keep myself from becoming physically unhealthy.*	.799 (.029)***						
**25.***I try to avoid engaging in behaviors that undermine my physical health.*	.759 (.032)***						
**35.***I really want to prevent myself from getting out of shape.*	.718 (.041)***						
**45***. I am motivated to avoid being in terrible physical shape.*	.715 (.042)***						
**16***. I am strongly motivated to devote time and effort to my physical health.*	.810 (.030)***						
**26***. I have a strong desire to keep myself physically healthy.*	.760 (.037)***						
**36***. It's really important to me that I keep myself in proper physical health.*	.724 (.045)***						
**33.***I usually worry about whether I am in good health.*	.565 (.057)***						
**4.***I feel confident about the status of my health.*		.728 (.050)***					
**14.***I rarely become discouraged about my health.*		.567 (.054)***					
**44.***I feel that I have handled my health very well.*		.737 (.041)***					
**9.***I expect that my health will be excellent in the future.*		.734 (.037)***					
**19.***I believe that the future status of my physical health will be positive.*		.757 (.049)***					
**10***. I am in good physical health.*		.805 (.030)***					
**20***. My body is in good physical shape.*		.786 (.041)***					
**2.***I sometimes wonder what others think of my physical health.*			685 (.055)***				
**12.***I’m very concerned with how others evaluate my physical health.*			.877 (.025)***				
**22.***I'm very aware of what others think of my physical health.*			.597 (.059)***				
**32.***I'm concerned about how my physical health appears to others.*			.871 (.027)***				
**42.***I'm concerned about what other people think of my physical health.*			.830 (.035)***				
**11.***I notice immediately when my body doesn't feel healthy.*				.824 (.029)***			
**21.***I'm sensitive to internal bodily cues about my health.*				.738 (.047)***			
**31*.****I know immediately when I'm not feeling in great health*				.761 (.047)***			
**41.***I'm very aware of changes in my physical health*				.782 (.043)***			
**17**. *My health is something that I alone am responsible for*					646 (.052)***		
**37.***What happens to my physical health is my own doing*					.798 (.035)***		
**47.***Being in good physical health is a matter of my own ability and effort*					.849 (.033)***		
**38.***Being in excellent physical shape has little or nothing to do with luck*					.531 (.072)***		
**48.***I don't believe that chance or luck play any role in the status of my physical health*					.533 (.069)***		
**3.***I feel anxious when I think about my health*						.755 (.036)***	
**13.***I'm worried about how healthy my body is*						.667 (.050)***	
**23.***Thinking about my health leaves me with an uneasy feeling*						.733 (.047)***	
**43***. I feel nervous when I think about the status of my physical health*						.749 (.049)***	
**39.***I will probably experience a number of health problems in the future*							.736 (.060)***
**49.***I anticipate that my physical health will deteriorate in the future*							.829 (.041)***

*MHPP* Motivation for health promotion and prevention, *HES* Health esteem, *HIC* Health image concern, *PHC* Personal health consciousness, *HLC* Health locus of control, *HA* Health anxiety, *HEX* Health expectation

*p<0.05

**p<0.01

***p<0.001

Table 4 reports Cronbach’s alpha for each of the seven factors and correlations among them. All factor displayed acceptable or good internal consistency. Significant correlations ranged between .248 and .737.

**Table 4:** Cronbach’s alphas and correlations among factors

Italian adaptation HOS	Cronbach’s alpha	MHPP	HES	HIC	PHC	HLC	HA	HEX
MHPP	.911	.						
HES	.888	.737***						
HIC	.876	.390***	.383***					
PHC	.857	.686***	.614***	.346***				
HLC	.815	.656***	.584***	.398***	.619***			
HA	.818	.401***	.180	.705***	.4327**	.363***		
HEX	.756	.248***	.148	.428***	.483***	.434***	.723***	

*p < 0.05, **p < 0.01, ***p < .001

*MHPP* Motivation for health promotion and prevention, *HES* Health esteem, *HIC* Health image concern*, PHC* Personal health consciousness, *HLC* Health locus of control, *HA* Health anxiety, *HEX* Health expectation

T-test was calculated to assess mean differences among smokers versus nonsmokers in the seven factors of the Italian adaptation of HOS. Nonsmokers reported higher values in Factor 1 MHPP [t(208) = − 2.739 *p* < .007] (CI 95% - 4.96 to −.809), Factor 2 HES [t(209) = − 3.387 *p* < .001] (CI 95% -3.93 to-1.03), Factor 3 HIC [t(213) = − 2.468 *p* < .014] (CI 95% -2.56 to −.28), and Factor 7 HEX [t(217) = − 3.451 *p* < .001] (CI 95% -1.45 to −.39) compared to smokers (see Table 5).

**Table 5:** Mean and standard deviation values and results of the t-test assessing differences between smokers and nonsmokers for Factor 1 (MHPP), Factor 2 (HES), Factor 3 (HIC) and Factor 7 (HEX)

Factors		M	SD.	T
Factor 1- MHPP	Smokers	25.14	8.01	t(208) = −2.739 **
	Nonsmokers	28.03	7.1	
Factor 2 - HES	Smokers	17.63	5.31	t(209) = −3.387**
	Nonsmokers	20.11	5.17	
Factor 3 - HIC	Smokers	7.21	3.92	t(213) = −2.468**
	Nonsmokers	8.64	4.29	
Factor 4 - PHC	Smokers	12.78	3.60	t(210) = .477
	Nonsmokers	12.56	2.95	
Factor 5 - HLC	Smokers	13.42	3.70	t(207) = −1.466
	Nonsmokers	14.12	3.14	
Factor 6 - HA	Smokers	7.37	3.37	t(211) = −1.419
	Nonsmokers	8.06	3.56	
Factor 7 - HEX	Smokers	5.62	1.92	t(217) = −3.451 **
	Nonsmokers	6.55	1.95	

*p < 0.05, **p < 0.01, ***p < .001

## Discussion

The last few years have been highly focused on health promotion programs, which aim to empower individuals and communities to choose healthy behaviors and make changes that reduce the risk of developing chronic diseases and other morbidities [74–76]. General public participation in their own health care decisions is increased [77–81]. Moreover, the recent approach of personalized medicine adopts a biopsychosocial dimension rather than a biomedical one. It starts with the assumption that the patient is a person, and not merely a body with an attached illness, and it supports patient empowerment and a shared responsibility between doctor and patient [77, 82]. In this framework it is pivotal to map psychological and cognitive aspects which could affect individuals’ attitudes in following health recommendations and guidelines for disease prevention [78, 79]. The degree of involvement in health-related decisions, as well as the amount of information desired, depends on the psychological, cognitive, social and cultural characteristics of each person [80].

The present investigation provided preliminary evidence for the validity of the Italian version of HOS. In details, results obtained from EFA and PA identified seven factors instead of 10 factors identified by Snell and colleagues. Fourteen items (1, 6, 7, 8, 18, 24, 27, 28, 29, 30, 34, 40, 46, and 50) were excluded, because they did not respect primary factor loading and secondary factor loading.

The first factor/subscale that we named Motivation for health prevention and promotion (MHPP) merged the two previous subscales on motivational tendencies (Motivation for healthiness and Motivation to avoid unhealthiness) and describes people that are strongly motivated to defend their well-being, to activate strategies which avoid risk behaviours that might compromise their health status and to adopt preventive behaviours. This style describes an active aptitude towards prevention. It also includes an item from the previous Health Anxiety scale since high concern and emotional reactions to the physical status might often affect motivation toward health-related behaviors or have significant negative impact on well-being [83].

Based on the self-determination theory – SDT [84] it is known that the level of individuals’ motivation affects the extent to which individuals will engage in, and persist with, health behaviors [85]. In particular, the autonomous motivation is an intrinsic tendency to achieve personal goals or outcomes, which the individual perceives as belonging to him/herself. Behaviors deriving from this kind of tendency are “self-determined”, and thus strongly elicited and sustained over time. Individuals motivated toward health behaviors feel a sense of choice, personal endorsement, interest, and satisfaction and, therefore, are likely to persist with the behavior.

The second subscale extrapolated by the factorial analysis was named the Health Esteem (HES), which describes people who show positive thinking and confidence in handling their health status, are optimistic about the future and perceive themselves in good physical shape. Studies in literature show that people who are confident about their health status and their future have more probability to follow a healthy lifestyle [38, 86], paying attention for instance to their diet, physical exercise and avoiding risk behaviours such as substance abuse [87].

Other two extrapolated factors reproduced the subscales Health Image Concern (HIC) and Personal Health Consciousness (PHC) of the original HOS scale. They describe respectively participants who might show apprehension regarding how other people could perceive their health status, their health image (HIC), and people who are a strongly aware and careful about their health status (PHC). People who endorse items belonging to the Health Image Concern subscale are usually moved by a kind of controlled motivation [84], an external pressure due to others’ opinion and worry for the rumours. They behave based on the impression they want to give and are more vulnerable to sudden behavioural changes. This style is often associated to a worse well-being, a poor quality of life and exacerbation of healthy behaviours turning into risky behaviours [88–90].

Finally, despite recent evidence show that health related behaviours may be activated even outside a conscious process, through for instance external stimuli able to change behaviour outside awareness [91], only higher degree of health consciousness guarantee higher willing to engage in those activities that are directly related to health [92, 93].

Two scales concerning locus of control were merged in the Italian version of HOS, with three items from the internal locus of control subscale and two items from the external locus of control subscale (“*Being in excellent physical shape has little or nothing to do with luck*” and “*I don’t believe that chance or luck play any role in the status of my physical health*”) which were considered a reverse in the Snell et al. version of HOS. Thus, people characterized by this kind of style overall believe that their well-being is under their responsibility, under their direct control (Health Locus of Control). Some studies in literature show that people who exert a control on their health have a positive thinking toward their health status and are able to modulate emotional distress caused by a disease, trying to face it actively with protective behaviors [94, 95]. For example, Kidd and colleagues [96] reported that patients with advanced coronary heart disease who had high levels of personal health control showed better outcomes in emotional reactions (e.g., depression reduction), physical activity, and quality of life after 3 months post-surgery (coronary artery bypass graft).

The last two factors extracted in the Italian adaptation of HOS scale identify people whose health perception is modulated by mood factors, such as worry and anxiety (Health Anxiety) and have negative expectation for their future health status (Health Expectation). These health orientation profiles are particularly important considering the current tendency in health psychology, which underlines the role of emotions in the modulation of health behaviours and health status [97]. For example, the *Broaden-and-Build Theory* by Barbara Friedrickson [98] argued that positive emotions facilitate thinking, action and decision-making. This approach suggests the importance in clinical practice of promoting positive emotions in patients. In the health domain, research suggests that global affective states – feeling good or bad – contribute to unhealthy behaviors such as smoking [94, 99–101], alcohol consumption [95, 102], and overeating [103]. Emotions also contribute to health-related risk perceptions [104], for example, worry about a health threat may trigger preventive behaviors [105].

Positive correlations among some of the seven factors extracted in the Italian adaptation of HOS seem to reveal that these subscales together express a “positive” and “active” attitude toward health promotion. For instance, Motivation for health promotion and prevention (MHPP) was strongly associated to Health Esteem, Personal Health Control and Health Locus of Control; Health Esteem was strongly associated to Personal Health Control, Health Locus of Control and Health expectation.

Other correlations, instead, revealed a more “anxious-emotional” approach toward health, such as Health Anxiety that was negatively correlated with Health Esteem, Health Expectation and positively correlated with Health Image Concern. It means that a higher emotional arousal toward the health corresponds to higher worry about the public impression of one’s own physical status, negatively affects self-esteem, but generates less negative expectation for the future, since it could turn to life style changes.

In order to replicate the factor structure and check the construct’s validity of the Italian adaptation of HOS, the scale was administered to a sample of tobacco cigarette smokers and nonsmokers. This study has a crucial importance, considering the incidence of the tobacco cigarette smoking in Italy both in adult and young population. World Health Organization (WHO) stated that until 2025 more than 19.7% of the Italian population will smoke, with a different distribution between male (23.3%) and female (16.3%) [106]. In addition, the study 2 provides pivotal suggestions in profiling and mapping psychological features of smokers and nonsmokers.

Results obtained by the Italian adaptation of HOS confirmed trends observed in Study 1. In fact, the factor structure of the HOS was replicated by analysis performed with CFA. Moreover, nonsmokers obtained higher value for “Motivation for Health prevention and promotion”, “Health esteem”, “Health image concern” and negative “Health expectation” compared to smokers. This means that nonsmokers reported a higher motivation to protect themselves from risk behaviours (such as cigarette smoking) or health damages, have a good feeling toward their health, are worried about their social image and have a realistic expectation about their future health status and the probability of incurring in health problems. Overall, we argue that these features might support the adoption of a better lifestyle, and may increase the motivation to adopt to preventive actions. Furthermore, smokers showed a lower motivation to adopt preventive actions to protect their health status and reported optimistic thinking about health status.

## Limitations

Despite the novel insight on relationship between personality characteristics and health behaviors, results should be treated with caution. The main limitation of Study 1 concerned data acquisition for the HOS questionnaire. In details, there were sporadic cases of *response set* and missing data probably caused by the high number of items in Snell and colleagues in the original version of the HOS (50 items) and the long time required to fill in the questionnaire. Another limitation concerned the channel used to disseminate the questionnaire. Indeed, for the questionnaire dissemination it was used mainly internet, we hypothesize that this might have introduced a possible *selection bias* achieving chiefly participants with a higher level of health literacy, and healthier lifestyles compared to the general population. Finally, only the construct validity was assessed according to our methodological plan.

This is a preliminary study along the validation process, in the next studies will be measured both predictive and convergent validity.

As well as the Study 2 reported two main limitations. First, the study is limited to smokers and nonsmokers, without considering former smokers who quitted successfully. In order to have a more detailed mapping of dispositional aspects that affect smoking behavior, future studies should enroll.

Secondly, participants were mainly *young adults* (mean age 31.47) with a low level of dependence (mean value 1.50). Future studies should overcome these limitations to allow a generalization of results.

## Conclusions

Summarizing, results obtained from the Study 1 and the Study 2 provide evidence on the ability of the Italian adaptation of HOS to distinguish dispositional and cognitive dimensions involved in health orientation styles. These and other evidence in the literature show how emotional, cognitive and behavioural tendencies are determinants of healthy behaviours, and how understanding patients’ psychological tendencies associated with health is fundamental in order to promote preventive health behaviours and to increase compliance with recommended health practices.

## Data Availability

The datasets generated and/or analyzed during the current study are not publicly available due protection privacy policy but are available from the corresponding author on reasonable request.

## Abbreviations

EFA: exploratory factorial analysis
HA: Health Anxiety
HCI: Health image concern
HEC: Health External Control
HEC: Health-Esteem and Confidence
HES: Health esteem
HEX: Health Expectation
HIC: Health Image Concern
HLC: Health locus of control
HOS: Health orientation scale
HS: Health Status
IHC: Health Internal Control
KMO: Kaiser-Meyer-Olkin
MAU: Motivation to Avoid Unhealthiness
MFH: Motivation for Healthiness
MHPP: Motivation for health promotion and prevention
PHC: Personal health consciousness
PHC: Personal Health Consciousness
